# Primary health care nurses’ management practices of common mental health conditions in KwaZulu-Natal, South Africa

**DOI:** 10.4102/curationis.v38i1.1168

**Published:** 2015-07-21

**Authors:** Faith N. Dube, Leana R. Uys

**Affiliations:** 1 Discipline of Nursing, School of Nursing and Public Health, University of KwaZulu-Natal, South Africa

## Abstract

**Background:**

Psychiatric conditions contribute to 13% of the global burden of diseases and account for one third of years lost because of disability (YLD). Despite the high prevalence of mental health problems, primary health care (PHC) services remain ineffective in managing patients with mental health conditions.

**Objectives:**

The aim of the study was to determine the practices of PHC nurses in the management of psychiatric patients in primary health care clinics in one of the rural districts in South Africa.

**Method:**

A survey was conducted amongst nurses working in several PHC clinics in KwaZulu-Natal (KZN) in order to determine their practices in the management of psychiatric patients. Mixed methods were used to determine the PHC nurses practices in the management of psychiatric patients.

**Results:**

The findings revealed that in five sites (83.3%) treatments are not reviewed every six months, there were no local protocols on the administration of psychiatric emergency drugs, and none of the study sites provided psychiatric patients with education on their medication and its possible side effects.

**Conclusion:**

Based on the results of this study it is evident that psychiatric patients at PHC clinics in the district where the study was conducted do not receive quality treatment according to institutional mental health guidelines.

## Introduction

Despite the high prevalence of mental health problems, primary health care (PHC) services remain ineffective in managing patients with mental health conditions. Psychiatric conditions contribute to 13% of the global burden of diseases and account for one third of years lost because of disability (YLD) (World Health Organisation [WHO] 2008a). An epidemiological study conducted in South Africa revealed that 16.5% of South African adults suffered from common mental disorders (Williams *et al.* 2008:4). The high prevalence of psychiatric disorders in South Africa can be attributed to exposure to violence and crime, the high prevalence of HIV infection and substance abuse (National Department of Health 2013b).

Prior to 1994, there was little consideration for primary mental health care and the policy of mental health services was to treat mentally ill people with medication and admission to psychiatric hospitals (Petersen *et al.* 2009). However as PHC clinics are generally the first point of contact patients seeking treatment (National Department of Health 2010), a mental health policy based on primary care principles was adopted in 1997 (WHO 2008b).

The mission for the South African National Department of Health is to ensure that all South Africans have access to good quality, affordable health care (WHO 2008b). Although the National Department of Health is responsible for developing mental health policy and guidelines, delivery of services is the responsibility of the provincial and district health departments (WHO 2008b). Therefore, with the promulgation of the *Mental Health Care Act* (17 of 2002) (National Department of Health 2002), it was the responsibility of provincial and district health services to integrate mental health services into PHC services (WHO 2008b).

In 2006, the National Department of Health released guidelines on the management of psychiatric patients at PHC level. The Standard Treatment Guidelines for Common Mental Health Conditions outline the following seven principles:

- Treatment begins with diagnosis, meaning a PHC nurse is obliged to take adequate history from the patient, relatives and other professionals who are treating the patient.
- Remember the person, meaning that any medical intervention should be made with the person as a whole in mind.
- Keep the treatment regimen simple so as to improve patient acceptability and adherence.
- Begin low, go slow, but reach high and long, meaning that the right dose of medication should be used for the correct duration.
- Antidepressants can be good for patients with any of the 10 common psychiatric diseases, such as schizophrenia, major depression and generalised anxiety disorder.
- Monitor side effects carefully.
- Consult when it is necessary (National Department of Health 2006).

However, despite the release of these guidelines, gaps still exist in the management of psychiatric patients in PHC clinics (Petersen *et al.* 2012) as de-institutionalisation was introduced without the necessary community based services, especially in rural areas (Department of Health 2013b). Petersen **et al.** maintain that psychosocial rehabilitation programmes are not provided in rural clinics, there is insufficient support for PHC clinic nurses in the management of psychiatric patients and poor identification and treatment of common mental disorders persist (Petersen *et al.* 2012). PHC services prioritise the control of chronic diseases that decrease life expectancy above diseases that cause disability (Grandes *et al.* 2011). Failure to diagnose mental health conditions may increase the rate of healthcare service utilisation (WHO 2008b).

Some psychiatric patients require continuity of care. They get psychiatric treatment every month from the PHC clinic and are reliant on the nurses who deliver the care. As a result of staff shortages in some PHC clinics, patients may be attended to by a registered nurse who has had no psychiatric training (Lund *et al.* 2008). The danger of this practice is that such nurses may misdiagnose a relapse in mental illness because of a lack of knowledge (Mwape *et al.* 2010).

### Problem statement

The National Mental Health Summit held on 12 and 13 April 2012 in Ekurhuleni, South Africa, was attended by representatives from government departments, non-governmental, research and user organisations, the WHO, academic institutions, professional bodies, traditional health practitioners, and clinicians. The Ekurhuleni Declaration on Mental Health (2012) that emanated during the summit concluded that integrating mental health services into primary health care, which is the foundation of the health care system, would increase prevention, screening, self-care, treatment and rehabilitation of patients with mental disorders and thus reduce the number of psychiatric patients referred for secondary and tertiary health care services. Even before the summit PHC nurses have been responsible for promoting mental health, detecting and treating people with common mental health conditions, ensuring an efficient referral system for patients requiring specialist care (Saxena *et al.* 2006) and providing follow-up treatment for patients who are stable (Petersen *et al.* 2009).

However, despite the strategies set out above, mental health in South African PHC clinics has a low priority and people with mental health disorders do not receive the care they deserve (Draper *et al.* 2009; Williams *et al.* 2008). Studies have found that nurses in PHC clinics are poor at detecting and managing mental health conditions (Naledi, Barron & Schneider 2011) and are not delivering psychosocial rehabilitation programmes, especially in rural clinics (Petersen *et al.* 2012).

There is shortage of human resources in PHC clinics which impacts negatively on integration of mental health into PHC. Awenva *et al.* (2010) maintain that one of the major challenges affecting the quality of mental health care services in PHC clinics is that primary health workers do not have the necessary time to provide quality mental health care. Furthermore, a South African study conducted by Van Deventer *et al.* (2008) revealed that PHC nurses do not have the necessary expertise to manage people with mental health conditions. Patients in the same study complained of seeing different health care workers every time they required follow-up treatment. Although psychiatrists should be available to supervise psychopharmacological care (National Department of Health 2013a), there are few available in the district where the research was conducted. There were only four full time psychiatrists based at the tertiary hospital providing specialist mental health services, which supports three districts with a population of 2 million (Department of Health 2014a). It was not clear how patients with psychiatric disorders were managed in the district.

### Significance of the study

Lack of support, training and supervision of health care providers to provide quality mental health care have been identified as contributing factors for poor detection of mental health conditions and poor management of people with mental health conditions (Naledi *et al.* 2011). As no previous studies have been conducted to investigate PHC nurses’ management of psychiatric clients in the context under study, the results of this study may contribute to improving the quality of care provided to people suffering from mental health conditions.

### Objective

The aim of the study was to determine the practices of PHC nurses in the management of psychiatric clients in PHC clinics in one of the rural districts in South Africa.

#### Research question

The research question was: ‘What practices are adopted and implemented by PHC nurses in the management of psychiatric clients at PHC clinics in one of the rural districts in South Africa?

#### Research method and design

A survey was conducted amongst nurses working in PHC clinics in a rural district in South Africa in order to determine their practices in the management of psychiatric patients. Mixed methods were used. For the qualitative aspect, semi-structured interviews, using an interview schedule with open questions, were conducted with PHC nurses. For the quantitative aspect, a questionnaire was used that included principles of supervision policy, which are continuity and accessibility.

Whilst some management issues were explored, the seven principles of the Standard Treatment Guidelines for Common Mental Health Conditions served as the basis for both the interviews and the questionnaire. However, principles 3 and 5 were excluded as nurses do not prescribe medication. Principle 4 was included because nurses monitor medication use and distribute the medication (see Table 1).

**TABLE 1:** Principles that were evaluated in the study.

Number	Principle
1	Treatment begins with diagnosis/adequate assessment
2	Remember the person
3	Simple treatment regime
4	Medication dose as low as possible
5	Antidepressants for anxiety
6	Monitor side effects
7	Consult when necessary
8	Continuity of care
9	Accessibility
10	Management of care

### Setting

The study was conducted in uThungulu Health District in the northern area of KwaZulu-Natal. This is the third largest district in the province with a population of 979 513, which is composed of Africans, white people and Asians (Department of Health 2013b). UThungulu district was selected as the site of the study because it represents a semi-urban and rural scenario (see Table 2).

**TABLE 2:** Classification and services in uThungulu District.

Local municipality	District hospital	Total number of PHC clinics	Number of clinics selected as cases	Number of psychiatric patients per month (average)
uMhlatuze (semi-urban)	0	*N* = 9	3	304
uMlalazi (rural)	3	*N* = 12	0	203
Mthonjaneni (rural)	1	*N* = 2	0	22
uMbonambi (rural)	0	*N* = 7	1	171
Ntambanana (deep rural	0	*N* = 5	1	53
Nkandla (deep rural)	2	*N* = 18	1	423

*Source*: World Health Organisation, (WHO), 2008b, *Integrating mental health into primary care: A global perspective*, World Health Organisation, Geneva

PHC, primary health care.

### Population and sampling

All clinics selected were public institutions providing the full PHC package as prescribed by the National Department of Health. The clinics were under the supervision of the hospitals attached to them in accordance with government recommendations that they be visited once a month by a PHC supervisor for mentoring and guidance (National Department of Health 2009). However, as a result of poor terrain which makes it difficult for the PHC supervisor to access them, the supervision rate for clinics in deep rural areas is 60%, which is far below the norm of 100% (Department of Health 2014a).

Whilst some clinics are semi-urban and therefore easily accessible, others are more rural, situated outside of towns or cities, where there is poor infrastructure. Those which are deep rural are at least 200 km away from urban areas and have extremely limited infrastructure and human resources (Department of Health 2014b). There were no urban areas in the health district in which the study was carried out.

Each clinic was visited on a separate date and had been advised of the visit. Convenient sampling was used whereby the researcher invited the health care workers present at the clinic to participate in the study. All health care workers were included, also those who were not registered as psychiatric nurses because it is possible that they would have to manage mental health patients because of limited staff. The sample therefore consisted of PHC nurses and staff nurses who were available during the visits.

The registered nurses in charge of the clinics (clinic managers) were purposely selected to complete the questionnaires as it required a self-report from them. Only 10 professional nurses and 5 staff nurses from six study sites from a total of 32 professional nurses and 15 staff nurses allocated to these clinics participated in the study as they were the only category of staff available during data collection at the clinics. Interviews were audio recorded for later transcription with participants consent.

Section three of the questionnaire involved reviewing patient records at the clinics. The criterion for inclusion of records was that the patient had to have been attending the clinic for three consecutive months without interruption. Records were randomly selected, and 110 of 415 met the criterion for inclusion. Entries in patients’ medical records were reviewed to assess the following:

- If patients’ medication had been reviewed in the six months prior to the study.
- If patients had been assessed before they were given treatment.
- If patients had been given education on their medication and possible side effects.
- If a medical examination had been conducted in the two years prior to the study.

Clear documentation was given a score of 1 and absence of data was scored 0. The average service utilisation for the clinics for three months, from October 2011 to December 2011 was noted.

## Research method and design

### Data collection process

The following instruments were used to collect data on the practices of PHC nurses in the management of psychiatric patients in primary health care clinics in one of the rural districts in South Africa:

- A questionnaire: The questionnaire was adapted from the PHC Supervision Manual for the purpose of collecting quantitative data from the registered nurse in charge (clinic manager) at each of the selected PHC clinics on the manner in which mental health conditions were managed at the particular clinic. The questionnaire had five sections with 24 items, 19 of which addressed the implementation practices in the management of psychiatric patients in PHC clinics. For the 19 items see Table 3.
- Record review: Section three of the questionnaire involved reviewing the previous three months’ clinical information recorded on clinic-held patients’ records. There were five items which addressed the clinical quality of mental health care provision to psychiatric patients.
- Interviews: An interview schedule with five semi-structured questions was used to collect data on the management practices of PHC nurses to determine how psychiatric clients were being managed.

The researcher collected the data, transcribed the recordings and analysed the data.

**TABLE 3:** Demographic profile of nurses who took part in the interviews (*N* = 15).

Qualification	Age Range	*N* = 15
General Nursing with Psychiatric Nursing	21–30	0
	31–40	0
	41–50	2
	51–60	1
Completed 4 year Diploma in Nursing	21–30	5
	31–40	0
	41–50	2
	51–60	0
Staff Nurse	21–30	3
	31–40	1
	41–50	0
	51–60	1

### Data analysis

Quantitative data were analysed according to simple descriptive analysis. Data were entered in the computer and analysed using SPSS (version 19) for Windows (spss-t2). A cross tabulation of the record review was carried out in order to ascertain whether there was any relationship between the geographical area and the management of the psychiatric patients.

Qualitative data were generated from the audio-recorded interviews by transcribing the interviews verbatim. Thematic analysis was used because of its benefit of flexibility (Braun & Clarke 2006). The researcher used Braun and Clarke's guide to the six phases of conducting thematic analysis which are: becoming familiar with the data, generating initial codes, searching for themes, reviewing themes, defining and naming themes, and producing the report.

## Ethical considerations

Ethical permission was sought from the University of KwaZulu-Natal Research Ethics Committee (reference number HSS/0653/011D) and from the KwaZulu-Natal Department of Health. Permission was also sought from uThungulu District Office Management and from the Chief Executive Officer of the hospital to which the clinics were attached. Participants were informed about the study and informed consent was obtained at the start of each clinic visit. Participants were informed that participation in the research project was voluntary. They were advised of the confidentiality and anonymity of their responses and that they could withdraw at any time without prejudice. The researcher conducted the interviews personally and ensured that the participants were at ease. A time for reflection was provided after the interview session.

Names for the interviewees were not used at any time. The responses from registered nurses were labeled as ‘RN1’ to ‘RN10’ and responses from staff nurses were labeled as ‘EN1’ to ‘EN5’. Data were coded by an independent coder to ensure trustworthiness.

### Reliability and validity

Data were triangulated from three data sources in order to facilitate comparative analysis of participants’ responses. This was carried out to ensure validity of the findings by cross checking the quantitative data with the qualitative data (Wilson 2014). Pattern matching was then performed in order to ensure internal validity (Voss, Tsikriktsis & Frohlich 2002).

## Results and discussion

Results from the questionnaire are summarised in Table 4 and the findings from the record review are summarised in Table 5.

**TABLE 4:** Results from PHC nurses in-charge (*N* = 6).

Number	Items	Response	Frequency	Percentage
1	Are the policies and guidelines on mental health and Act available?	Yes	2	33.3
		No	4	66.7
2	Are services available daily: Known psychiatric patients?	Yes	4	66.7
		No	2	33.3
3	Are services available daily: People in emotional crisis?	Yes	6	100
4	Are services available daily for people requiring counselling?	Yes	6	100
5	Are services available daily for mental health care users referred from hospital to the PHC facility?	Yes	6	100
6	Does a mental health team visit this facility to consult and attend to referred patients?	Yes	1	16.7
		No	5	83.3
7	Are records of attendance of psychiatric patients kept to enable follow-up of defaulters?	Yes	4	66.7
		No	2	33.3
8	Are written referral criteria and routes available?	No	6	100
9	Could a client in a current crisis receive continuity of care from the same practitioner?	No	6	100
10	Are people with symptoms of mental illness, given a full physical examination including neurological system, blood glucose, signs of substance abuse?	No	6	100
11	Is medication available to patients who require medication on PHC EDL?	Yes	6	100
12	Does this facility take routine blood from patients requiring monitoring, e.g. patients on lithium, clozapine, tegretol, etc?	Yes	6	100
13	Is emergency treatment for side effects available?	Yes	5	83.3
		No	1	16.7
14	Are there local protocols on the administration of psychiatric drugs for psychiatric emergency?	Yes	1	16.7
		No	5	83.3
15	Is it possible for a psychiatric patient to see the same health worker, to provide continuity of care?	No	6	100
		No	6	100
16	Are there support groups for patients and their families?	Yes	4	66.7
		No	2	33.3
17	Are written annually updated referral protocols in line with the mental health care available?	Yes	4	66.7
		No	2	33.3
18	Is feedback received, informing the PHC facility of their role in continuation of care?	Yes	4	66.7
		No	2	33.3
19	Are written signed emergency treatment/management protocols for psychiatric patients in place?	Yes	3	50
		No	3	50

**TABLE 5:** Record review according to geographical area (*N* = 110).

Variable	Geographical area and frequency			Percentage
	Semi-urban	Rural	Deep rural	
**Age of patient**				
15–25	2	10	1	11.8
26–35	11	10	6	24.5
36–45	11	15	6	29.1
46–65	16	7	12	31.8
66 +	1	0	2	2.7
**Gender categories**				
Female	19	13	19	46.4
Male	22	29	8	53.6
**Marital status**				
Married	5	4	8	15.5
Single	36	38	18	83.6
Widowed	0	0	1	0.9
**Employment history**				
Employed	3	1	0	3.6
Unemployed	38	41	27	96.4
Educational status				
Primary	10	16	4	27.3
Secondary	15	15	2	29.1
None	16	11	21	43.6
**Questions, Yes/No**				
Has the medicine required been reviewed in last 6 months?				
Yes	2	0	0	1.8
No	39	42	27	98.2
Has a medical exam been carried out in the last 2 years?				
Yes	0	0	1	.9
No	41	42	26	99.1
Has an in-depth interview been carried out in last 2 months?				
No	41	42	27	100
Was education given in last 6 months?				
No	41	42	27	100
Was education on side effects given?				
No	41	42	27	100

### Demographic information of nurses who completed the questionnaire

Six female registered nurses in charge of the six clinics completed the questionnaire. Three of the registered nurses were aged between 51 and 60 years, one was between the ages of 31 and 40 and one between the ages of 41 and 50. Two of the nurses had a four year nursing diploma, one had a diploma in psychiatric nursing and three had general nursing only.

### Demographic profile of nurses who took part in the interviews

Interviews were conducted with 10 registered nurses and 5 staff nurses. All nurses who took part in the interviews were female. One registered nurse was aged between 51 and 60 years and had a qualification in General Nursing with Psychiatric Nursing; four were between 41 and 50 years, two of whom had a qualification in General Nursing with Psychiatric Nursing and two had completed a four year Diploma in Nursing; and five were between 21 and 30 years and had a four year Diploma in Nursing. One staff nurse was aged between 51 and 60 years, one was between 31 and 40 and three between 21 and 30 (see Table 3).

### Demographic profile of record review

The 110 records reviewed revealed that the highest percentage (97.4%) of patients was treated in rural clinics as compared to semi-urban (1.8%) and deep rural areas (0.9%). Most patients from semi-urban (*n* = 36) and rural (*n* = 38) study sites were single. Statistics showed no significant difference in unemployment rate between semi-urban (*n* = 38) and rural areas (*n* = 41). The educational status was low in deep rural areas, with 21% of participants being recorded as having no education (see Table 5).

#### Findings from the questionnaire

The findings revealed that policies and guidelines on the *Mental Health Care Act* (17 of 2002) were not available in 66.7% of the clinics surveyed. Participants indicated that patients in emotional crisis, patients requiring counselling and patients referred from hospitals were attended to daily in all the study sites.

Written referral criteria and routes were not available in any of the study sites and a client in a current crisis could not receive continuity of care from the same practitioner. People with symptoms of mental illness were not given a full physical examination, including an examination of the neurological system, blood glucose tests and assessment for signs of substance abuse. Medication was available to patients as on the PHC Essential Drug List (EDL) and routine monitoring of drug levels was carried out in all study sites. Sixty seven percent of study sites did not have support groups for people with mental health conditions. Feedback informing the PHC facilities of their role in continuation of care was only received by 66.7% of the clinics.

## Results from interviews

Themes relating to the practices of PHC nurses were generated based on the interview schedule that was used to collect data. The results are reported according to the themes. Data from the survey and the record review have also been integrated into each theme.

### Theme 1: Treatment begins with diagnosis

During interviews nurses indicated that psychiatric patients are given a full physical examination, including blood glucose tests as evident in the excerpt below:

’We also assess them the mental symbol whether he is well oriented, is he relapsing, some of them are having fits, assess how often do the fits occurred, how are they, how do they look. Are they oriented, are they responding or not responding.’ (RN1)

This was not consistent with the findings of the document review which indicated that people with symptoms of mental illness had not been given a full physical examination which included the neurological system, blood glucose tests and assessment for signs of substance abuse in any of the study sites (Table 4).

Findings revealed that in-depth interviews had not been held with patients at two month intervals as prescribed, but that critical information had been evaluated when the patient came to collect treatment. This was confirmed by one of the nurses who said:

‘Though it is a summary but you do that general appearance of a client, do orientation, memory as you remember [RN1]. But I have always seen summary being done.’ (RN5)

### Theme 2: Remember the person

Participants indicated that patients who are seen to be restless in the queue are attended to first:

’We do fast-track those we know cannot wait.’ (RN5)

In some clinics, interviewees indicated that patients are treated with courtesy by greeting them before they are attended to.

### Theme 3: Medication dose as low as possible

In 98.2% of the cases, the record review revealed that medicine required by the psychiatric patients had not been reviewed in the six months prior to the study (Table 5). It was observed in the patients’ records that some patients were receiving the same treatment that had been prescribed six years previously. There was no evidence whether patients had been given education about their treatment in the six months prior to the study.

### Theme 4: Monitor side effects

Nurses in all study sites indicated that they take blood samples from patients whose drug levels need monitoring. This was confirmed during the interviews:

’Bloods are taken in six months and they then go to the doctor.’ (RN6)

Whilst emergency treatment for side effects was available in five of the study sites, it was not available in one site.

### Theme 5: Consult when necessary

Referral criteria and routes were not available in the clinics where the study was conducted and it is therefore not clear what procedures are followed when PHC nurses refer patients to the next level of care. During interviews participants indicated that they refer patients who are due for treatment review and monitoring of lithium levels. They also indicated that they consult the psychiatric trained nurse who is based at the hospital psychiatric clinic when they are not sure of what to do:

‘… Then we consulted, and ask about bloods and about policies, what is needed so as to have direction of what to do in order to improve care of psych.’ (RN2)

Nurses indicated that they also consult other stakeholders, such as police officers, if they are faced with a patient who is violent. Patients with a first episode of mental illness and patients who show signs of aggression are transported to the hospital with the help of police officers, if necessary:

‘If a new patient coming from home telling us that she has delusions for the first time, we first check whether she is on any treatment. If he is too violent, we give him injection so that he becomes calm and we call an ambulance. If he becomes way beyond the ambulance we call the police and they do respond.’ (RN1)

### Theme 6: Continuity of care

Table 4 shows that nurses in all six study sites indicated that it is impossible for a patient in crisis to receive continuity of care from the same practitioner. This was affirmed by one of the nurses who said:

‘It's not one sister who attends to them. It's like daily allocation. If I am not in someone else do attend to them …’ (RN1)

To ensure continuity of care, nurses recommended that:

‘There must be a person who must be solely responsible for them so that she can even attend workshops and be updated with new things that are happening in psych.’ (RN3).

Nurses in four of the sites (66.7%) indicated that continuity of care is a challenge because they do not receive feedback when a patient has been referred to hospital.

### Theme 7: Accessibility of care

Nurses at all the study sites reported that services for people in emotional crises and those requiring counseling were available on a daily basis as well as services for psychiatric patients who had been referred to PHC from hospital. However, services for psychiatric patients were only available daily in four of the sites (66.7%). Patients attending the other two sites (33.3%) were seen by a psychiatrically trained nurse on a dedicated day once a month. One of the interviewees said:

‘When they come to the clinic, they come in groups. We attend to them in the morning when they come. We have a nurse who attends to them every first Friday of the month.’ (RN)

The participant added, however, that if the dedicated registered nurse is off duty on that day, another professional nurse is allocated to attend to patients:

‘… But if I am not in someone else do attend to them.’ (RN)

In the clinics where services are available on a daily basis, patients join the general queue and are seen by the registered nurse who happens to be on duty on that day:

‘They are attended by the sister who is available. If we see there is a problem, we refer.’ (RN10)

This is consistent with the findings of the questionnaire where the nurses in charge at all study sites (100%) indicated that patients are seen by whichever nurse is on duty on the day rather than by the same nurse on subsequent visits (Table 4). Participants indicated that psychiatric patients are promptly attended to if they become restless whilst in the queue:

‘There are two or three clients who do not want to sit down. So you just check them and not make him to wait.’ (RN5)

### Theme 8: Management of care

The findings revealed that mental health guidelines, including the *Mental Health Care Act* (17 of 2002), were not available in four of the study sites (66.7%). Furthermore, five (83%) of the study sites were not visited by the mental health team. Only one site reported being visited once a month by the mental health team from the mother hospital.

Records of attendance to enable tracing of patients defaulting treatment were not available in four sites (66.7%). Nurses put the blame on the system for introducing integration of service:

‘Since the integration of mental health was implemented, and it was said the clinic must be every day, Aii!, you see that is where we started to have defaulters.’ (RN6)

They expressed that it was not easy to identify patients who had not come for treatment:

‘Like now as they are coming at any time, you are not able to see who has come, who has not come. If you have specific day you will know that you have seen all 10 patients.’ (RN8).

There were no local protocols on the administration of emergency psychiatric drugs in five of the sites (83.3%). Moreover, none of the study sites had support groups for psychiatric patients and their families or referral protocols. One of the interviewees acknowledged the fact that they are not managing the psychiatric patients properly by saying, ‘We have not been well established in attending to them the correct way. But we have just started to take care of them’ (RN1).

### Theme 9: Problems impacting on quality mental health provision

In four of the study sites (66.7%) staff had received in-service education provided by the mother hospital, but none of the study sites had programmes of in-service training on mental health planned for the year. This was consistent with the findings of the interviews where some of the nurses indicated that they needed in-service education:

‘But we need a person who will update us and tell us what is expected so that patients can get total nursing care. Most of the nurses last have the information during their training and we are not sure what to do.’ (RN2)

Nurses indicated that they do not provide quality mental health because they experience shortages of staff:

‘Due to shortage of resources, we tend to do things routinely. We summarise as I have just said that we are now doing summary.’ (RN5)

## Discussion

The study explored the practices used in the management of psychiatric clients at selected PHC clinics by looking whether PHC nurses implemented the principles of the Standard Treatment Guidelines for Common Mental Health Conditions. The discussion is in accordance with the principles.

### Principle 1: Treatment begins with diagnosis

Although psychiatric patients should be given a full physical examination which includes assessment of the neurological system, blood glucose and signs of substance abuse before a diagnosis are made, the results revealed no evidence that any thorough assessment or any psychosocial interventions were being implemented. This could be attributed to PHC nurses’ lack of knowledge and skills with respect to psychiatric patients, or, as in a study conducted by Petersen *et al.* (2009) where nurses verbalised that assessing a psychiatric patient needs extra time.

None of the study sites had a programme for in-service training on mental health although such training would enable PHC nurses to provide quality mental health care and thus meet the medical and psychological needs of the patients (Collins *et al.* 2010). Saraceno *et al.* (2007) recommend that nurses in PHC facilities be provided with training in mental health to enable them to assess and treat mental health problems.

Training and capacity building of PHC nurses must be an ongoing process because new nurses who have not been trained in mental health are continually being allocated to PHC facilities.

### Principle 2: Remember the person

The Patients’ Rights Charter (Department of Health 2001) clearly states that patients have a right to privacy and confidentiality. In a study conducted by the WHO (2008b:155), in Ehlanzeni District in Mpumalanga Province, South Africa, 99% of nurses interviewed felt that patients with mental illness should receive the same care as any other patient in the PHC setting. The findings of the current study revealed that patients in all of the study sites were treated with respect and dignity. Each patient was afforded time to be alone with the health care provider in the consulting room.

The PHC clinics have a duty to establish support groups for psychiatric patients and their families, but it was found that there were no support groups for psychiatric patients and their families in any of the sites. Offori-Atta, Read and Lund (2010) are of the opinion that support groups would assist families in caring for their family members suffering from mental health conditions.

### Principle 3: Start medication dose as low as possible

The patients’ treatment was not monitored. For a person with a psychiatric condition, the need to take medication for a long time may affect compliance (Serobatse, Du Plessis & Koen 2014).

### Principle 4: Antidepressants for treatment of anxiety

This principle was excluded as nurses do not prescribe medication and treatment for newly diagnosed patients it is prescribed by the doctor.

### Principle 5: Monitor side effects

Side effects were not monitored by nurses (Table 4) and education on side effects was not given. This is consistent with the findings of the study conducted by Hetrick *et al.* (2011) who found that nurses only became aware that patients were experiencing side effects if this was brought to their attention by these patients. The care provided at the clinics seems to be dictated by routine, based only on providing medication.

### Principle 6: Consult when necessary

Findings revealed that nurses did seek advice when they need guidance in the management of psychiatric patients, but this consultation was not guided by any criteria. PHC nurses are not responsible for initiating treatment (Petersen *et al.* 2009).

### Principal 7: Continuity of care

Findings showed that there were variations on how psychiatric patients were being managed in the different study sites. In some clinics, services for psychiatric patients were offered daily, whilst in others, services were only offered once a month on a dedicated day. Patients attending the clinics which offer a daily service joined the general queue and were seen by whichever nurse was on duty at the time. There is, therefore, no continuity of care. Clients, health care workers and policy makers consider continuity care as an effective strategy in the management of long term psychiatric disorders (Burns *et al.* 2009). The findings of the study conducted by Green *et al.* (2008) revealed that continuity of care was associated with good recovery and better quality of life.

## Limitations of the study

One of the limitations identified in this study was that it did not investigate whether the patients were satisfied with the service they received from the study sites. According to Westaway *et al.* (2003), a satisfied patient is free to utilise the health service and comply with the treatment plan. They advise that patient satisfaction can be measured by getting to know the patients’ experiences regarding health care.

## Recommendations

The researcher recommends that PHC mental health services should be scaled up by offering services that are based on scientific evidence and by adhering to available mental health guidelines and protocols (Eaton *et al.* 2011). Psychosocial rehabilitation at clinic level should be strengthened to ensure that psychiatric patients are managed holistically (Petersen *et al.* 2009).

It is also recommended that mental health care in primary health care clinics be revived through retraining of PHC nurses on mental health care. Mentoring and supervision of PHC nurses should be carried out so that they provide quality mental health services (Saraceno *et al.* 2007). In terms of improving the mental health care, it is recommended that support groups for psychiatric patients and their relatives be established because they not only provide a recovery-oriented treatment that is not provided through medication, but also help to reduce stigma (Goldstrom *et al.* 2006). Psychosocial rehabilitation programmes should be implemented in PHC clinics as part of management of psychiatric patients (Petersen *et al.* 2012).

## Conclusion

Based on the findings of the study, it is evident that psychiatric patients do not receive the quality mental health services they deserve. The study identified a lack of knowledge and skills amongst PHC nurses as contributing factors to the poor management of psychiatric patients in the study sites. Staff shortages in PHC clinics are a barrier towards provision of quality mental health care, especially in rural clinics because these clinics are overburdened with multiple programmes and high patient workloads (Sareceno **et al.** 2007). It became apparent that principles 1 (assessment), 4 (lowest dose) and 6 (management of side effects) are not being adhered to in PHC clinics. This is the result of both a lack of adequate preparation of the nurses doing the work, lack of support by other health care providers, such as psychiatrists, as well as the lack of adequate guidance on the guidelines provided.
